# Reading Notices

**Published:** 1910-09

**Authors:** 


### READING NOTICES.

### THE HAY-FEVER PROBLEM.

Again the physician is called upon to grapple with hay-fever, and a veritable army of sneezing, watery-eyed "miserables" come to him for relief. For a long time the idea was prevalent that little or nothing could be done for these people. The patient dreaded the coming of the disease, and the physician dreaded the coming of the patient. The situation was one of ample misgivings and scanty faith. Now it ispretty well recognized that medication, while still empiric to a certain extent, is nevertheless effective. The symptoms can be controlled or greatly minimized, and the patient may have the relief he seeks. And for this much he will be truly thankful, and the physician, in turn, duly thanked.

Adrenalin is perhaps the most effective agent. It antagonizes the symptoms and secures to the patient a marked degree of comfort. It allays the congestion of the mucous membrane, reduces the swelling of the turbinal tissues, controls the nasal discharged, cuts short the violent paroxysms of sneezing and the abundant lacrimation, and prevents depression by stimulating the heart.

The practitioner who desires to employ Adrenalin in the treatment of hay-fever has recourse to the product in a number of forms. Adrenalin Chloride Solution (1:000) is doubtless the most widely used. It is first diluted with four to five times its volume of physiological salt solution, then sprayed into the nares and pharynx. Adrenalin Inhalant has many adherents. This is an oil solution, and is administered by spray. It may be diluted

Page(s) missing

